# Thermoanalytical approach to assess riverine PET litter and its recycling potential

**DOI:** 10.1038/s41598-025-94925-y

**Published:** 2025-05-05

**Authors:** Ferenc Ronkay, Emese Slezák, Dániel Gere, Nóra Lukács, Miklós Gyalai-Korpos, Attila Dávid Molnár, Katalin Bocz

**Affiliations:** 1 Jászberény Campus, Eszterházy Károly Catholic University, Rákóczi út 53, Jászberény, 5100 Hungary; 2 Imsys Engineering Services Ltd., Mozaik Street 14/A, Budapest, 1033 Hungary; 3 Department of Organic Chemistry and Technology, Faculty of Chemical Technology and Biotechnology, Budapest University of Technology and Economics, Műegyetem rkp. 3, Budapest, 1111 Hungary; 4 Department of Polymer Engineering, Faculty of Mechanical Engineering, Budapest University of Technology and Economics, Műegyetem rkp. 3, Budapest, 1111 Hungary; 5 Department of Hydraulic and Water Resources Engineering, Faculty of Civil Engineering, Budapest University of Technology and Economics, Műegyetem rkp. 3, Budapest, 1111 Hungary; 6 Technology and Innovation Unit, Plastic Cup Society, Gutenberg tér 2. V/3, Szolnok, 5000 Hungary; 7 River Monitoring and Citizen Science Unit, Plastic Cup Society, Gutenberg tér 2. V/3, Szolnok, 5000 Hungary

**Keywords:** Riverine litter, Circular economy, PET bottles, Thermal analysis, Environmental ageing, Mechanical recycling, Environmental sciences, Chemistry, Engineering, Materials science

## Abstract

In this proof-of-concept study, a thermoanalytical method based on differential scanning calorimetry (DSC) is proposed to quantitatively link the crystallisation dynamics of bottle-grade poly(ethylene-terephthalate) (PET) with its degradation state. The study uniquely simulates long-term sunlight exposure through controlled artificial ageing of PET bottles in a Xenon chamber, with degradation levels accurately determined via intrinsic viscosity (IV) measurements. Following degradation, DSC analysis was conducted, and the complex melting endotherms observed during the post-isothermal crystallisation heating phase were deconvoluted into sub-peaks using the Fraser-Suzuki function. A novel discovery was made: a specific sub-peak exhibited a melting temperature with a strong, linear relationship to the molecular chain length of PET, providing a precise indicator of polymer degradation. This correlation represents a significant advancement in the field, as it was successfully applied to PET litter samples collected from floodplains in Hungary within the catchment area of the Tisza River, enabling accurate estimation of environmental degradation levels. Moreover, based on the determined IV ranges of the riverine PET bottles, we propose targeted recycling strategies for this pervasive pollutant, with a comprehensive evaluation of reprocessing outcomes. These findings open new avenues for assessing polymer degradation in environmental and industrial contexts, highlighting the method’s potential for broader applications in waste management and material science.

**Supplementary Information:**

The online version contains supplementary material available at 10.1038/s41598-025-94925-y.

## Introduction

The Danube River Basin, spanning 19 countries, is a textbook example of the far-reaching consequences of transnational plastic pollution waves. Although significant data gaps exist concerning riverine litter and plastic pollution, research in the Danube River Basin is advancing considerably to tackle the magnitude of the issue at both microplastic^1^ and macroplastic^2^ levels. The Danube’s longest tributary, the Tisza River drains an area of approximately 157,000 square kilometres, flows through several countries, including Ukraine, Romania, Hungary, Serbia, Slovakia, and Moldova, and transports large quantities of plastic pollution into the European Union.

The primary driver of increasing plastic pollution in rivers is inadequate waste management infrastructure, with waste collection and transportation non-existent in at least 196 municipalities in the upstream sections of the Tisza^3^. In recent years, climate change has also become a significant contributing factor, as flash floods and intensified surface runoff have caused substantial damage to property and infrastructure^4^. These extreme weather events have led to more frequent and severe waves of plastic pollution, worsening the environmental and economic impacts on aquatic ecosystems^5^. According to moderate estimates, 4.2 tons of plastic are discharged daily into the Black Sea^2^. However, monitoring efforts involving water authorities figures^3^, remote sensing-^6^, and citizen science data^7^ indicate that only a fraction of this pollution reaches the lower stretches of the Danube and, eventually, the Black Sea. The rest gets deposited in the alluvial floodplain forests, with more than 1665 tons of plastic waste already deposited in the floodplains of the Tisza River alone^3^.

As plastic degradation transforms alluvial macroplastic accumulations into secondary microplastics, it becomes increasingly imperative to manage the largest deposits^8^. While beach cleanup operations as well as river cleanups, can be costly and necessitate additional funding^5^, the development of a marketable circular raw material derived from collected riverine plastics presents an opportunity to advance the circular economy. This approach not only facilitates the recycling of materials but also contributes to habitat restoration in alluvial ecosystems.

According to the reports of the European Union, about 80–85% of the accumulated beach waste is plastic^9^, almost half of which is single-use product waste^10^. Comparing this value with the composition of the Tisza litter collected in Hungary in recent years (Figure S1), it can be seen that the mass ratio of plastics, in this case, is much less (about 15–37 wt%), but not negligible; and the contribution share of PET as a single-use beverage bottle is exceptionally high. The research so far shows that noticeable thermal, photochemical, or hydrolysis-related degradation processes may occur on poly(ethylene terephthalate) (PET) products that have spent a longer time in nature; the materials exposed to the greatest stress and damage are those where light and moisture are present at the same time^11^. These conditions can be met quite easily in the case of plastics stuck in river floodplains, making the degraded material nearly impossible to recycle, which is why collecting waste as early as possible and properly sorting it is a crucial task. In the case of PET waste collected from natural waters, an additional problem can be that individual bottles spend different periods in watery conditions, therefore it is difficult to determine the degradation of the material set, the quality of incoming bottles can vary by area, collection period, and even from bottle to bottle. Although the amount of waste and its composition in the Tisza catchment area have been monitored since 2004^12^, no detailed study has been published on the quality analysis of plastics. To be able to integrate these materials back into the cycle, it is necessary to know the rheological properties important from the point of view of processing and thus to be able to find a solution for the potential areas in which the available raw material of variable quality can be reused. Thus, before recycling, it is necessary to assess which waste fractions are already too degraded to be recycled and which can still undergo the ecologically preferred mechanical recycling.

From the point of view of PET processing and reprocessing, the molecular weight (M_w_) is a key property. While Gel Permeation Chromatography (GPC) is the gold standard for measuring molecular weight distribution, intrinsic viscosity (IV) is more commonly used due to its simplicity and lower cost. The IV value, related to the polymer’s average molecular weight via the Mark-Houwink equation^13^, can indicate degradation. However, both GPC and IV methods require hazardous solvents, and the equipment is less common in industry. Alternatively, Melt Flow Rate (MFR) provides an indirect indication of viscosity in the molten state of PET, influenced by temperature, pressure, and processing conditions, but IV remains a more reliable measure of molecular weight and end-use properties.

In the literature, there are some examples of how the degradation of PET can be investigated in addition to the IV value with measurement methods that are more common in practice. Among others, PET degradation has been examined using Fourier transform infrared spectroscopy (FTIR)^14^, X-ray diffraction^15^, viscometric measurements, end group analysis by titration^16,17^, and nuclear magnetic resonance spectroscopy (NMR)^18^ as well.

Some of these analytical methods were used for the modelling of environmental degradation and ageing studies as well. Ioakeimidis et al.^19^ studied PET bottles collected from the Aegean Sea using the ATR-FTIR measurement method and utilizing the expiration dates on the bottles to determine the age of the samples. Based on their tests, PET bottles remain robust for about 15 years, after which the changes in peak intensity allowed them to infer structural changes. The results were later compared with the measurement results of samples from the Ionian Sea. Using the method, they were able to give fairly accurate predictions (± 3 years) regarding the expiration date of PET bottles from the given sea.

However, in most cases, the exact date when the item was released into the environment is not known, so it is difficult to track changes over time. In most research studies, the degradation of the material is induced under artificial, well-regulated conditions, thereby modelling the processes taking place in nature. Wu et al.^20^ investigated the ageing of PET in a marine environment for substantial and artificially accelerated ageing samples. The influence of various environmental effects on the mechanical properties, viscosity, and thermal and chemical composition of the samples was investigated. Based on the results, the degradation of marine PET was mostly influenced by light and wet environments. Although the salt present had little effect on degradation, it was able to penetrate the structure of the material and could not be effectively removed by simple physical cleaning.

Gok et al.^21^ investigated the degradation of PET films with different PET stabilizers using four accelerated weathering arrangements, including prevalent stresses like UV light, heat and freeze exposure, and humidity. The tests were carried out over seven weeks, while samples for testing were taken every week. The samples’ yellowness index (YI), opacity (%), and degradation were followed using FTIR, and changes in the optical absorbance of the degraded films were analysed using UV-Vis spectroscopy. The FTIR tests indicated plentiful scissions in the polymer over UV exposure. They also found that the yellowing was mostly caused by UV light exposure, while hazing was observed in the case of high humidity. The environmental effects on the material were more severe when light and moisture were present simultaneously. UV stabilization protected the polymer during the first stage of exposure, but later on, a linear increase in yellowness and opacity was observed.

Recently, a differential scanning calorimetry (DSC) approach was proposed for assessing the quality of polyethylene terephthalate (PET) waste of various origins and quality^22^. The basis of this concept relies on the correlation between the molecular structure (i.e. M_w_ distribution, type, and ratio of comonomers) and the thermophysical properties of the PET crystals and crystallisation kinetics^23–25^. Šudomová et al.^22^ applied statistical methods and utilized the correlations of various parameters obtained by DSC analyses to distinguish and categorize PET waste samples. This method proved to be applicable to analyse the samples qualitatively, but quantitative assessment e.g. on the degree of degradation, cannot be made with this approach.

Upon heating following the isothermal crystallisation of PET, the multiple melting endothermic peak appearing on the DSC curve is a phenomenon that has been observed and researched for more than 30 years^26–29^. In the early literature, three types of sub-peaks were generally observed, which were named I, II, and III sub-peaks in the temperature order of their formation, and their origin was explained by different recrystallisations and lamella thickenings taking place during the heating phase of the study. Ronkay et al.^30^ did not group the sub-peaks in order of their temperature of appearance, but according to their morphological properties, thus identifying a total of five sub-peak clusters. The crystallisation time, temperature, and molecular weight dependence of each sub-peak cluster were determined. At high temperature (460 K <) crystallisation, where the molecular chains have higher thermal energies, no. 4 and no. 5 of these were reported. The behaviour of the sub-peak set 4 fits the Strobl model^31^. As the crystallisation time increases, it progressively transforms into sub-peak 5 during recrystallisation. The sub-peak no. 5 is probably the result of the crystallisation of pre-organised mesomorphic particles to form crystalline layers and lamellae. The part of sub-peak no. 4 that has not been converted to no. 5 during isothermal crystallisation remains unstable and recrystallises during the DSC heating test, thus appearing on the melting curve with a melting point higher than no. 5.

The average molecular weight (M_w_) affects the thickness of the formed crystalline layers of sub-set no. 5, and thus their melting temperature (T_m_), which is a precisely measurable parameter. Considering this, in this research study, our hypothesis was that after isothermal crystallisation, the determination of T_m_ of the sub-peak no. 5 is a suitable method for indirectly estimating M_w_ for PET samples of various origins. Knowing the M_w_ range, the product properties can be designed, or possible value-adding modifications can be adjusted.

## Materials & methods

### Materials

#### Original PET (OPET)

NEOPET 80 type PET (Neogroup, Lithuania) with an intrinsic viscosity of 0.80 ± 0.02 dL/g and a melting temperature of 284 ± 4 °C was purchased for the artificial ageing tests.

#### Riverine PET recyclate (RPET)

Bottles were collected from the river basin of Tisza, from the banks of Bodrog River between Olaszliszka and Bodrogkisfalud (Hungary) as part of the annual multiple days cleanup competition on Bodrog River, the Bodrog Plastic Cup^12^ in August 2023. Volunteers of the Plastic Cup initiative were approaching the polluted floodplains by canoe from the river to collect the samples. The previous monitoring actions helped them to find the polluted areas and know where to land on the banks. The bottles were collected randomly from the ground of the floodplain forest. Bottles from four different sampling locations were tested, the coordinates of which were: 48°14’16.2 “N, 21°26’40.6"E; 48°13’38.4"N 21°25’20.7"E; 48°12’11.6"N 21°24’22.6"E and 48°10’44.2"N 21°22’12.3"E (see also in Figure S2).

Bottles collected from floodplain forests were washed with water at 65 °C containing 1% NaOH to remove labels and mud. The washing was performed under laboratory conditions at an agitation speed of 300 rpm for 15 min, with 10 PET bottles washed at one time in 40 l of washing liquid (i.e., an approximated plastic-to-water ratio of 1:100). Then, smaller pieces were cut out from the sidewall of 15 randomly selected bottles for the DSC and IV analyses, while 5 kg of bottles were shredded and homogenized during the mechanical recycling process.

### Sample preparation

#### Blow-moulding

Bottles with 5 l capacity were blow-moulded from the NEOPET 80 material with an ASB-70DPH (NISSEI ASB MACHINE CO., Japan) single-stage industrial blow moulder. The melt temperature was 260–275 °C, the injection speed was 150 mm/s, the cooling time was 30 s, the mould temperature was 12 °C, and the holding pressure was 80 bar during the injection moulding of the preform. The following parameters were applied for the bottle blowing: the preheating temperature was 110 °C, and the blowing pressure was 30 bar.

#### Compounding and injection moulding

The riverine PET flakes were first ground using an SM 300 mill (Retsch, Germany) to make it easier to feed during extrusion. After that, the polymer was dried for 4 h at 160 °C in a hot air dryer (Memmert, Germany) to reduce its moisture content. The water content of the dried material was measured to be 74 ppm using a Hydrotracer FMX (Aboni, Germany) device in accordance with the ISO 15512:2019 (E) standard^32^. Extrusion took place using an LTE 26–48 (Labtech Scientific, Italy) twin-screw extruder with an L/D ratio of 48 at a screw speed of 80 rpm. The barrel temperatures ranged from 255 to 265 °C, and the die pressure was 37–52 bars. The extruded granules were then dried again for 4 h at 160 °C. After that, ISO dumbbell specimens were injection moulded according to the ISO 294-1:2017 (E) standard^33^ using an ES 200/45 HL – V hydraulic machine (Engel, Austria). The barrel temperatures varied from 270 to 285 °C, the screw speed was 480 rpm, the holding pressure was 70–75 bars. The die temperature was set to 45–55 °C, and the cooling lasted 20–27 s.

### Characterisation methods

#### Ageing

The blow-moulded bottles were aged in a Q-Sun XE-3-HS (Q-LAB, USA) Xenon chamber with a planar sample holder. 5 cm^2^ pieces were cut out from the bottles, and a Daylight-Q filter with a minimum wavelength of 295 nm was applied to mimic the radiation of the Sun. The test was carried out in the form of 120-minute continuous daylight exposure at 50% relative humidity, followed by an 18-minute light and water spray cycle. The temperature of the black panel was set to 63 ± 5 °C, and the airspace of the chamber was at 38 ± 3 °C. The irradiation intensity was 60 W/m^2^ in the wavelength range of 300–800 nm, while the slope of the table was 10°. One ageing cycle consisted of 1 week (168 h), and the samples were removed for DSC and intrinsic viscosity tests. The overall ageing lasted for seven weeks (1176 h), which means 254 MJ/m^2^ energy in the UV range (300–400 nm), which is approximately more than a year of direct sunshine^34,35^.

#### Intrinsic viscosity (IV) determination

An RPV-1 (PSL Rheotek, UK) Ubbelohde viscometer with 1B capillary was applied for the intrinsic viscosity (IV) measurements of the bottles. As a reference, NEOPET 80 granules were tested, as well. The specimens were dissolved in 60:40% phenol – 1,1,2,2-tetrachloro-ethane at 100 °C for 1 h. The concentration of the tested solution was 0.5 g/dL. The test was completed at 30 °C, following the ASTM D4603:2018 standard^36^. The results were calculated from the average of two parallel measurements. Based on a previous study conducted by Ronkay et al.^30^, the Mark-Houwink constants were calculated, and the average molecular weight of the samples could be determined by Eq. (1).

1 $$\:\left[\eta\:\right]=1.10\cdot\:{10}^{-3}\cdot\:{\left({M}_{w}\right)}^{0.64}$$

where $\:\left[\eta\:\right]$ is the intrinsic viscosity [dL/g], and M_w_ is the weight-average molecular weight [g/mol]. Based on a similar principle, the number-average molecular weight can be calculated, too, according to Eq. (2).

2 $$\:\left[\eta\:\right]=1.64\cdot\:{10}^{-3}\cdot\:{\left({M}_{n}\right)}^{0.66}$$

where M_n_ is the number-average molecular weight [g/mol].

Dispersity, defined as the ratio of the two molecular weights, provides information on the molecular weight distribution, and it is expressed as Eq. (3):

3 $$\:\rlap{--} D=\frac{{M}_{w}}{{M}_{n}}$$

where Đ is the dispersity or heterogeneity index [-].

#### Differential scanning calorimetry (DSC)

**Fig. 1:**
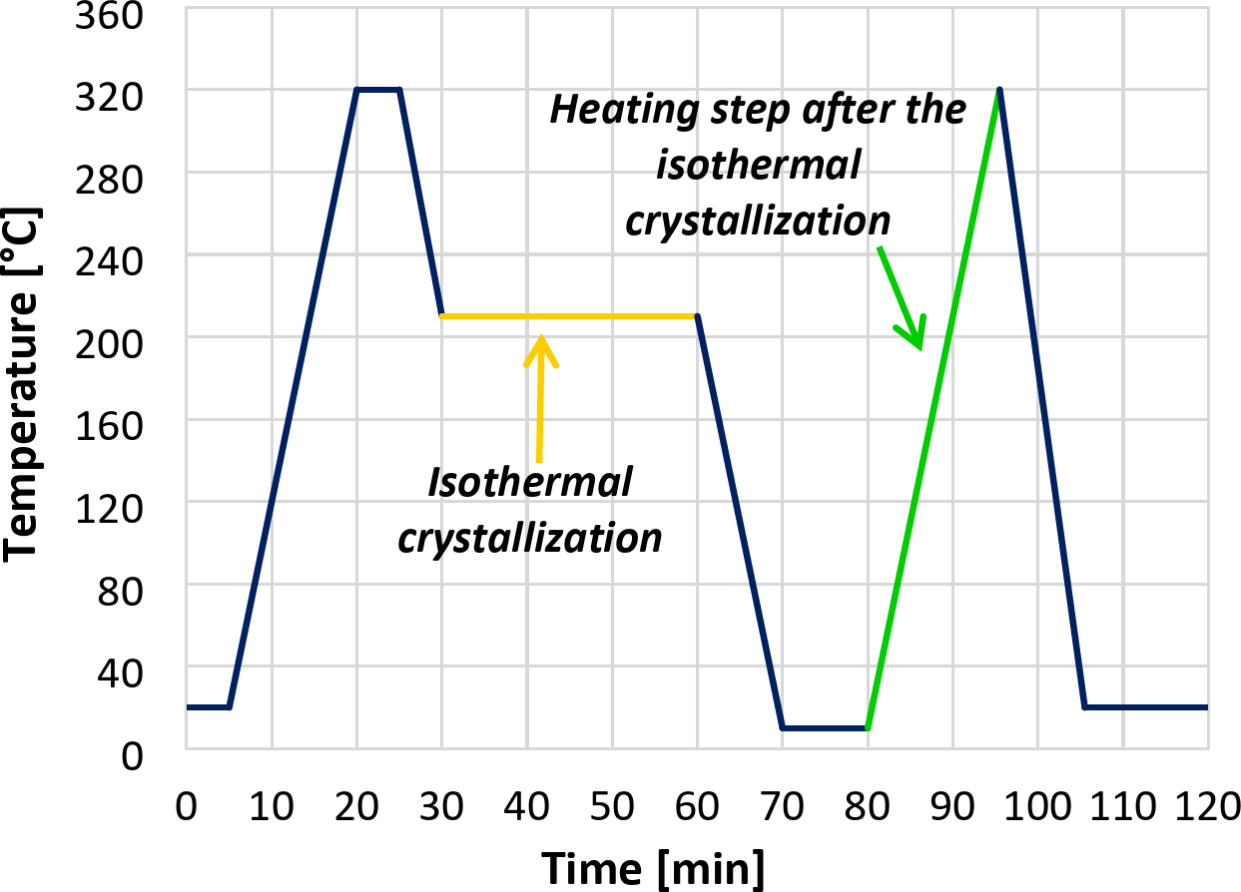
Schematic presentation of the DSC program.

The isothermal crystallisation process was conducted using a DSC 131 Evo (Setaram, France) differential scanning calorimeter (DSC). The specimens were tested in a nitrogen atmosphere, and the cooling/heating rate was set to 20 °C/min. After stabilizing the temperature at 20 °C for 5 min, the specimens were heated from 20 to 320 °C, kept at 320 °C for 5 min, then cooled to 210 °C to the temperature of isothermal crystallisation. The temperature was kept constant for 30 min, then the specimen was cooled to 10 °C, remained at 10 °C for 10 min, and heated to 320 °C again. The program ended with uncontrolled cooling to room temperature. The visual representation of the DSC program can be seen in Fig. 1. The ratio of crystallinity was calculated with Eq. (4):

4 $$\:{\chi\:}_{c\:}\left[\%\right]=\frac{{\varDelta\:H}_{m}-{\varDelta\:H}_{cc}}{\varDelta\:{{H}_{m}}^{0}}\cdot\:100\%$$

where χ_c_ is the crystalline ratio of the sample [%], ΔH_m_ is the melting enthalpy of the sample [J/g], ΔH_cc_ is the enthalpy of cold crystallisation [J/g], ΔH_m_^0^ is the melting enthalpy of a perfect crystal (140 [J/g] for PET^37^). The mean and standard deviation of the measurements were calculated from two replicates.

To calculate the crystallisation rate, first, a tangential-sigmoid baseline was fitted to the isothermal crystallisation curve, then a conversion function following the crystallisation process was applied. The crystallisation time corresponding to the 50% conversion - half-time crystallisation (t_1/2_) - was determined, and the crystallisation rate was interpreted as the reciprocal of the t_1/2_ in seconds. An example of the half-time crystallisation calculation is presented in the Supplementary Material (Figure S3).

#### Tensile testing

A 3369 (Instron, USA) universal mechanical tester was used for the tensile tests conducted on the injection moulded specimens. The gripping distance was 115 mm, and the test speed was 50 mm/min. The test was done according to the ISO 527-1:2019 (E) and ISO 527-2:2012 (E) standards^38,39^.

#### Izod impact testing

A 5115 10/01 (Zwick, Germany) impact tester was applied for the Izod impact tests. A 2 mm, V-shaped notch was created on the injection moulded specimens with an RM2125 RT (Leica, Germany) rotation microtome. Ten specimens were tested from each composition in accordance with the ISO 180:2023 (E) standard^40^. The pendulum’s nominal energy was 5.5 J, and the frictional loss was 0.033 J.

## Results and discussions

### Qualification tests for artificially aged PET bottles

**Fig. 2:**
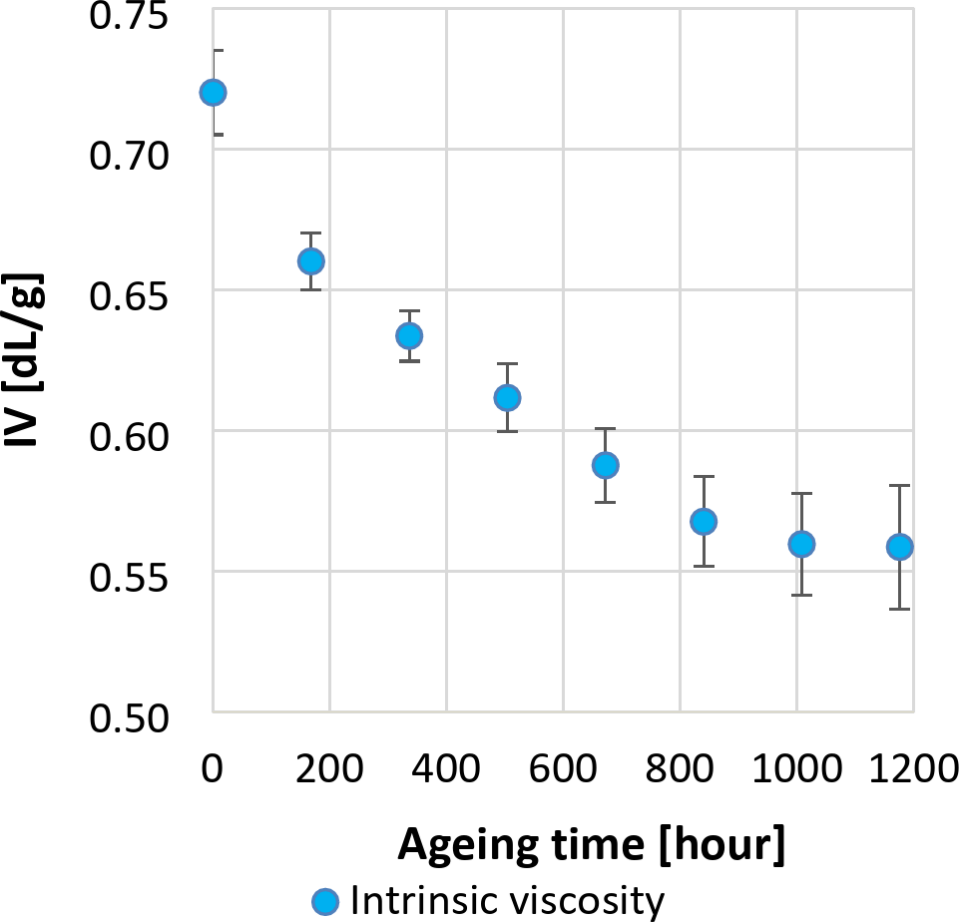
Intrinsic viscosity of aged PET bottles.

To explore the relationship between the crystallisation dynamics of bottle-grade PET and its degradation state, the degradation process of newly manufactured PET bottles subjected to accelerated weathering stresses of UV light and humidity was first monitored by IV measurements. It can be seen in Fig. 2 that the IV value of the original PET granulate decreased to IV = 0.72 ± 0.02 dL/g during bottle production when the material was subjected to mechanical and thermal stress. Then, the IV value decreases gradually over UV irradiation time. Since the temperature set in the Xenon chamber (38 °C) was below the glass transition temperature of PET, it can be assumed that the decrease in IV is predominantly related to the UV irrradiation induced cleavage of polymer chains. The shorter chains formed during photodegradation are characterized by greater mobility accompanied by lower viscosity. The evolution of number-average and weight-average molecular weights, as well as dispersity, can be observed in Table 1. The moderate change in dispersity over the aging period can be attributed to the competing effects of chain scission and cross-linking during artificial aging. In the early stages, chain scission dominates, with longer chains breaking more readily, resulting in a narrower molecular weight distribution. As aging progresses, cross-linking reactions become more significant, particularly among shorter chains, leading to a broader molecular weight distribution. Over aging time, the balance between these two processes stabilizes dispersity.

**Table 1:** Calculated molecular weights (number and weight average) and dispersity of artificially aged PET samples.

Ageing time [h]	Number average molecular weight [g/mol]	Weight average molecular weight [g/mol]	Dispersity [–]
0	10,087	25,113	2.49
168	8,841	21,921	2.48
336	8,309	20,561	2.47
504	7,876	19,457	2.47
672	7,412	18,277	2.47
840	7,033	17,314	2.46
1008	6,884	16,934	2.46
1176	6,865	16,887	2.46

**Fig. 3:**
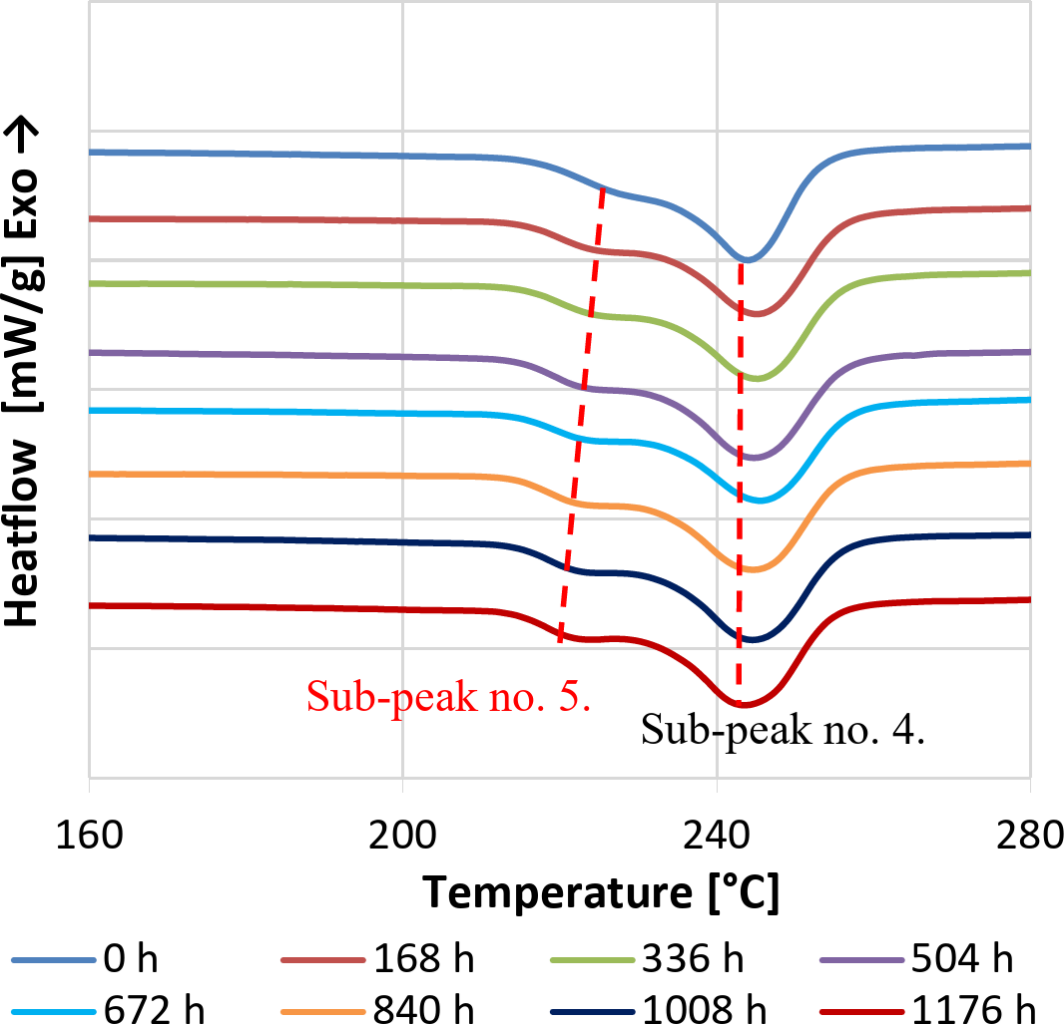
DSC heating curves after the isothermal crystallisation at different UV exposure times.

**Fig. 4:**
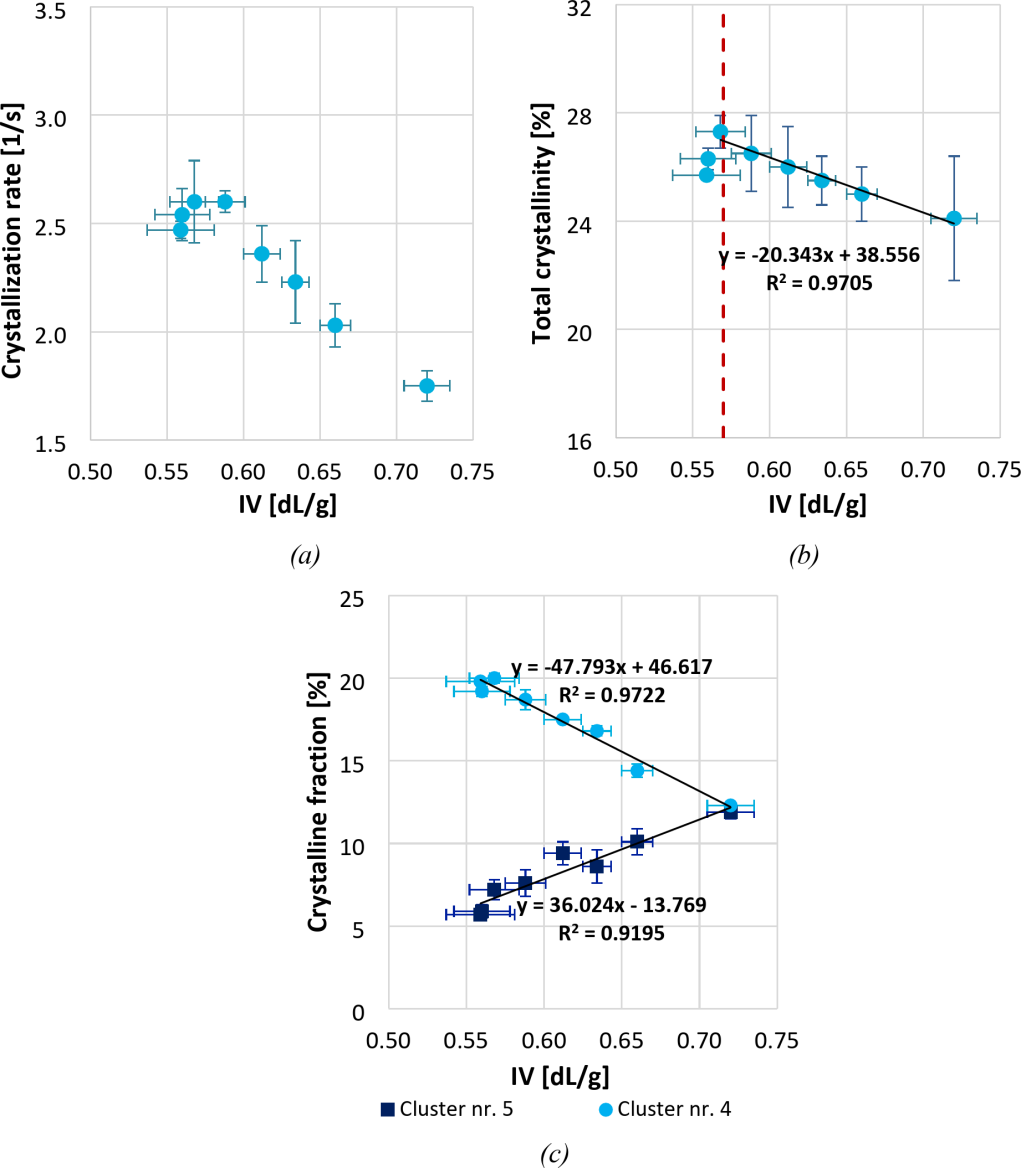
(**a**) Crystallisation rate and (**b**) total crystalline fraction detected during the heating after isothermal crystallisation as a function of intrinsic viscosity and (**c**) crystalline fraction corresponding to sub-peaks 4 and 5.

Samples taken weekly from the aged PET bottles were subjected to isothermal crystallisation at 210 °C and subsequent heating ramp in the DSC apparatus (according to the program presented in Fig. 1) to analyse the melting endotherms. The heating DSC curves showed double endothermic peaks, which were identified according to literature^30^ as cluster no. 4 and no. 5, respectively. In Fig. 3, the changes in the peak areas and shifts in the peak temperature of the melting endotherm no. 5 as a function of exposure time are clearly visible. Evaluating the thermograms it was established that initially, with the ageing time, the reduction of the IV value is associated with an increase in the crystallisation rate (Fig. 4a) and the crystalline fraction (Fig. 4b). The correlation between the IV value and the total crystallinity is linear with a good approximation in a wide IV region; below the IV value of about 0.57 dL/g (which corresponds to an estimated M_w_ of 17,433 g/mol), however, the crystallinity begins to sharply decrease with further reduction of the IV. These observations are consistent with the literature that, in general, the crystallisation rate and the crystalline fraction increase with decreasing molecular weight, but this trend does not hold for lower molecular weight ranges^41,42^. According to the results obtained from aged PET samples shown in Fig. 4b, the degree of crystallinity increases as the molecular weight increases for molecular weights below 17,433 g/mol. For this reason, the IV value cannot be reliably determined over a wide range from the crystallisation rate or crystallinity. A separate analysis of the endotherms seems to be more appropriate. For this purpose, the DSC curves were fitted with a tangential sigmoid baseline, then the double endothermic peaks were separated into two sub-peaks using the Fraser-Suzuki function^30^ capable of handling the possible asymmetry of the recorded curves, and the fitting parameters were determined. The fitted DSC curves and the separated peaks are presented in the Supplementary materials (Figure S4). The crystalline fractions associated with each sub-peak are presented in Fig. 4c. It can be seen that in the case of increasingly degraded samples, the proportion of the crystalline part connected to the sub-peak set no. 5 decreases, while that of the subset no. 4 increases. These results support previous findings^29,43^, according to which, in the case of crystallisation above 190 °C, the primarily formed preordered mesomorph parts with increasing IV are more able to transform into crystalline lamellae. As the cluster fraction increases, the lamella thickness, and hence the melting temperature, shows an increasing trend.

**Fig. 5:**
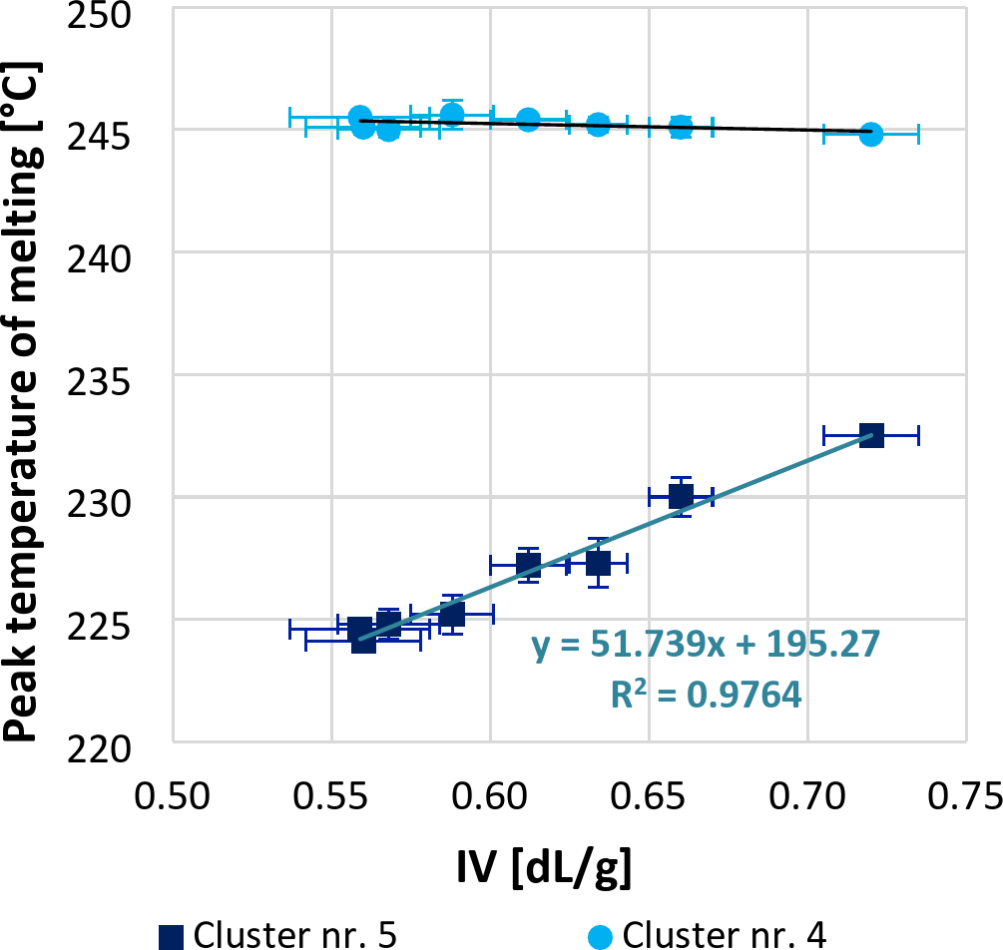
Peak temperature of melting measured during the heating after isothermal crystallisation as a function of intrinsic viscosity.

In Fig. 5, the melting temperatures recorded for the individual sub-peaks are plotted as a function of IV. It can be seen that the temperature of the sub-peak no. 4 is not significantly affected by the IV change. In contrast, the melting temperature of the sub-peak no. 5 (T_m5_) is significantly influenced by the length of the molecular chains; the more degraded the material is (so the lower the IV value), the lower the temperature of the crystal set connected to the sub-peak no. 5 melts, which indicates lower lamella thickness. The linear relationship between T_m5_ and IV of the polymers appears to exist over the entire investigated, wide IV range, even in the low-molecular-weight region. The correlation between T_m5_ and IV is given by Eq. (5), and is considered valid at least in the M_w_ range of 16,887 to 25,113 g/mol. It was concluded that T_m5_ detectable after the isothermal crystallisation of PET can be a suitable parameter for estimating the IV value of various PET samples. It should be noted that while calculating enthalpy, the experimentally obtained value of ΔH_m_ is strongly influenced by the chosen baseline^44^. However, the construction of the baseline does not affect the melting peak temperature, therefore the accuracy of determining the melting peak temperature is higher than that of crystallisation rate or crystallinity, allowing for a more precise property estimation.

5 $$\:IV=\frac{{T}_{m2}-195.3}{51.74}$$

### Use of the method for the analysis of riverine PET waste

PET bottle samples recovered from the Bodrog River were subjected to DSC runs while the melting endotherms were analysed according to the newly elaborated method (Figure S5). For each sample, T_m4_ and T_m5_ values were determined from the heating curve after isothermal crystallisation at 210 °C, besides, IV measurements were done using the standard solvent-based method. In the next step, the IV values (IV predicted) were calculated from the T_m5_ values determined from the Bodrog samples using the same equation (Eq. (5)), and then this value was compared to the actual measured IV values of the samples. This was considered as the absolute error of the model according to Eq. (6).

**Fig. 6:**
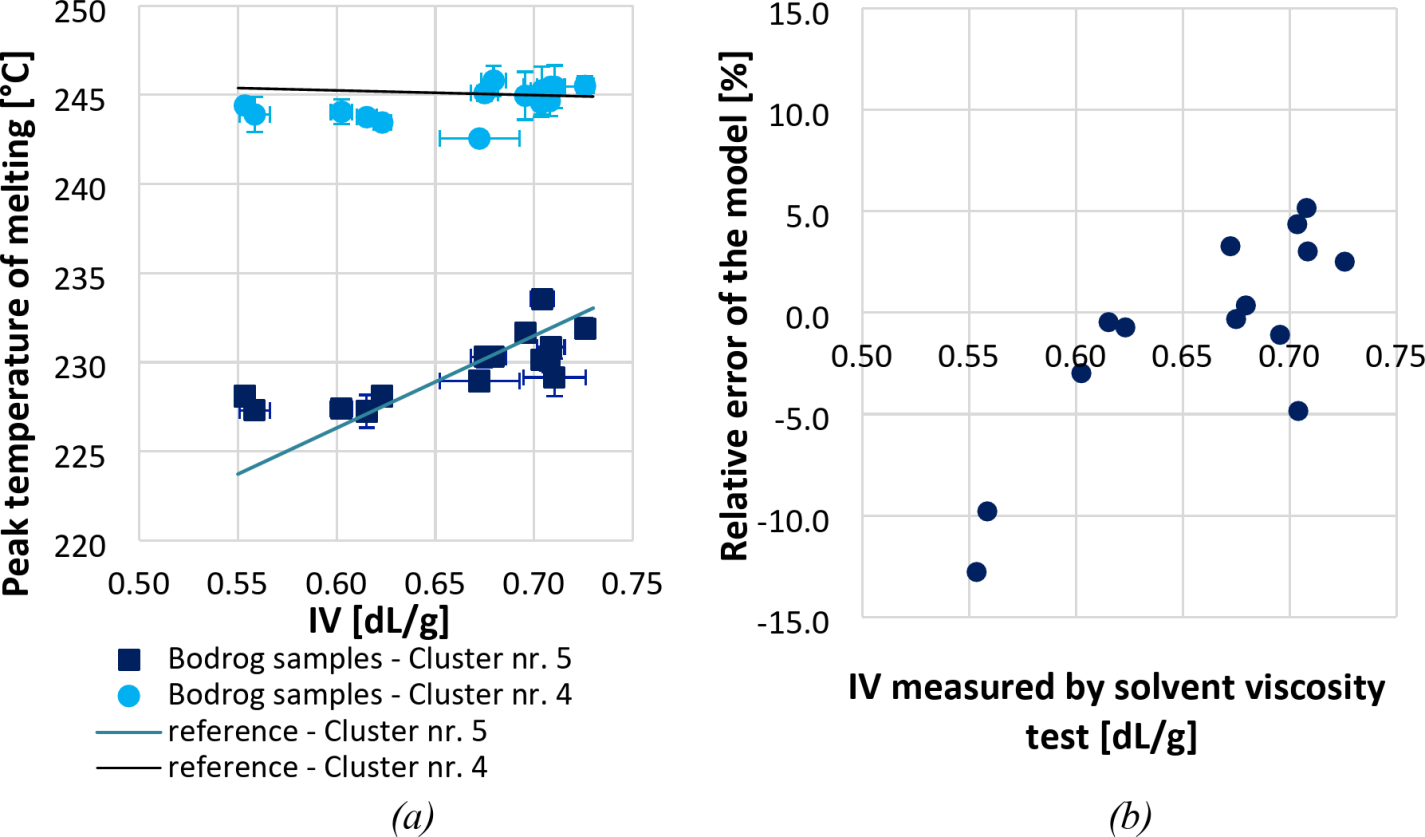
(**a**) Peak temperature of melting of sub-peaks 5 and 4 for samples collected from Bodrog and (**b**) Difference between the measured and the predicted IV.

The obtained melting temperature versus IV value pairs of riverine PET bottles are plotted in Fig. 6a, together with the linear regression lines fitted to the artificially aged PET bottle samples (see Fig. 5). It can be seen that the T_m4_ values of the riverine PET samples fall into a range (vary around 245 °C) with the corresponding values of the model material. Also, the T_m5_ values of the riverine bottles, being increasingly sensitive to changes in the length of the molecular chains, fit well to the established correlation equation (Eq. 5), nevertheless, in the lower IV region (below 0.60 dL/g) the T_m5_ values deviate towards slightly higher values than expected based on the actually measured IV values. This anomaly may be attributed on the one hand to the potential presence of comonomers, catalyst residues, or various additives (such as pigments, plasticisers, fillers, chain extenders, etc.), which can affect the crystallisation characteristics of PET^45,46^. On the other hand, the artificial aging conditions used provide only relative results and cannot perfectly simulate the slow natural degradation processes. While hydrolytic degradation primarily leads to chain scission, photo-oxidation initiated by UV light can also induce crosslinking and alter the molecular structure, affecting the crystallization behavior of the polymer. Nevertheless, even with these distorting effects, the revealed correlation can be well exploited to estimate the degradation state of the waste bottle samples.

For evaluation of the estimation accuracy of the method, the IV values estimated based on the DSC analyses, as described by Eq. (5), were compared with the actually measured IV values. For this purpose, first the absolute error of the model was calculated according to Eq. (6), which is the difference between the measured and the predicted IV:

6 $$\:Absolute\:error={IV}_{measured}-{IV}_{predicted}$$

Then, the absolute error was applied to calculate the relative error of the model, which is essentially the absolute error divided by the estimated value, as shown by Eq. 7:

7 $$\:Relative\:error=\frac{Absolute\:error}{{IV}_{predicted}}$$

It can be seen in Fig. 6b that the relative error of the model is lower than ± 6% in the IV range of 0.60 to 0.73 dL/g, (i.e. in the M_w_ range of 18,888 to 25,660 g/mol), and it still remains lower than ± 13% even in the lower viscosity (< 0.60 dL/g) range. This ± 6% accuracy in the most relevant IV range is comparable to that of IV measurements for PET samples, which, given proper procedural adherence to the ASTM D4603 (E) standard, typically ranges from ± 1% to ± 3%. MFR determinations also have an accuracy of ± 1% to ± 5% under optimal conditions, as per ISO 1133-1:2022 (E) standard^47^. However, in practice, the error of these measurements is often higher.

Overall, our estimates indicate that the proposed thermoanalytical method provides reliable molecular weight information of various PET samples at a significantly lower cost per sample than traditional IV measurements and in an environmentally friendly manner.

### Characterisation of mechanically recycled riverine PET

As plastic litter gradually accumulates on riverbanks and then flushes away during heavy rains or floods, the degradation state of the PET bottles may largely vary on the location and timing of the litter collection. The IV of the examined PET bottles collected from the Bodrog River in 2023 falls in the range of 0.55–0.73 dL/g, which reflects a broad range of degradation states for the recovered riverine plastic.

Typically, mechanical recycling is the preferred method for recycling PET products. This involves collecting and shredding used PET items into small pieces, followed by washing and removing contaminants before melting the material to create PET regranulate. Clear and blue bottle flakes with minimal degradation and an IV value near 0.72 dL/g can be processed into food-grade materials after undergoing solid-state polycondensation (SSP) and super cleaning processes^48^. It is commonly believed that mechanical recycling is advisable for waste with an IV value of approximately 0.70 dL/g or higher^49,50^ as the ecological footprint of mechanically recycling more degraded PET waste would be excessively large; and therefore, chemical recycling may represent the optimal solution in such cases^51,52^.

In this study, as a first approach, the mechanical recyclability of riverine PET waste was investigated without any quality-based separation or value-adding modification. Mechanical tests were performed on injection moulded specimens obtained after melt-compounding the mixed shredded flakes of the recovered bottles of various physical states. An IV value of 0.58 ± 0.1 dL/g was measured for the obtained recycled extrudate indicating significant degradation and loss of the rheological properties during reprocessing. This IV closely approximates the most degraded PET bottles found among the riverine litter. The mechanical characteristics of the recycled riverine PET (rrPET) material are presented in Table 2 together with those of original PET (oPET) and industrially recycled food packaging (selectively collected bottles from municipal source) PET material (rPET)^53^. It can be seen that despite degradation from environmental exposure and reprocessing, the main mechanical properties, such as tensile strength, tensile modulus, and notched impact strength decreased by less than 10% compared to the original PET. A noticeable difference was only observed in the elongation performance, which characteristic shows increased sensitivity to the molecular weight. Our results indicate that based on its comparable strength, stiffness, and impact behaviour the mechanically recycled riverine PET is a suitable material for the preparation of various plastic products.

**Table 2:** Mechanical characteristics of original and recycled riverine PET and industrial recycled PET.

	Tensile strength [MPa]	Elastic modulus [MPa]	Elongation at break [%]	Notched Izod impact strength [kJ/m^2^]
oPET (IV = 0.80 dL/g)	58.6 ± 3.5	2220 ± 50	48 ± 9	3.9 ± 0.2
rPET (IV = 0.75 dL/g)^25^	59.8 ± 0.3	2114 ± 13	15 ± 9	3.5 ± 0.2
rrPET (IV = 0.58 dL/g)	54.5 ± 4.2	2090 ± 42	4 ± 1	3.6 ± 0.4

## Conclusion

Plastic litter must be caught in rivers before it ends in seas and oceans, and the largest possible share must be returned to circulation by mechanical recycling technologies. Knowing the viscosity of the recovered material is essential in terms of thermomechanical processability. This research study introduces a new thermal analysis-based method for accurately estimating IV necessitated for assessing the quality and potential recyclability of riverine PET litter. Our findings demonstrate that the melting temperature of sub-peak no. 5, determined after isothermal crystallisation using a DSC apparatus, is appropriate to predict the molecular weight of various PET samples, including environmental litter. The DSC-based method aligns with the principles of green chemistry by eliminating the need for hazardous solvents and provides a significantly lower cost per sample compared to traditional IV measurements.

The total amount of PET litter which is removed only from the Tisza catchment area amounts to an average of 12.4 tonnes per year^12^. Through the reintegration of collected riverine PET waste, which is currently not sorted for mechanical recycling, back into circulation, both society and the environment would benefit. Collecting and utilizing riverine waste helps preserve clean water by reducing microplastics, protecting the oceans, and supporting the protection of terrestrial ecosystems by alleviating the burden on landfills. Our study posits that even unseparated riverine PET litter fractions are suitable for mechanical recycling, yielding recycled products with decent mechanical properties. Nevertheless, to enhance the reintegration of PET litter into circulation, implementing quality-based separation of the collected material for customized recycling could be essential. Additionally, improving the properties of recycled plastics through methods such as toughening or reinforcement could significantly boost their performance and usability as durable, high-performance materials for technical applications.

## Electronic supplementary material

Below is the link to the electronic supplementary material.

Supplementary Material 1

## Data Availability

The data that support the findings of this study are available from the corresponding author upon reasonable request.
