# Supplementary material for: Thermoanalytical approach to assess riverine PET litter and its recycling potential
Source: Sci Rep. 2025 May 5;15:15673. doi: 10.1038/s41598-025-94925-y (PMC12053600; doi:10.1038/s41598-025-94925-y)
Supplement: Supplementary file 1 — Supplementary Material 1 [file 41598_2025_94925_MOESM1_ESM.pdf]

Thermoanalytical Approach to Assess Riverine PET Litter and Its Recycling Potential

Supplementary Materials

Ferenc Ronkay, Emese Slezák, Dániel Gere, Nóra Lukács, Miklós Gyalai-Korpos, Attila Dávid Molnár, Katalin Bocz*

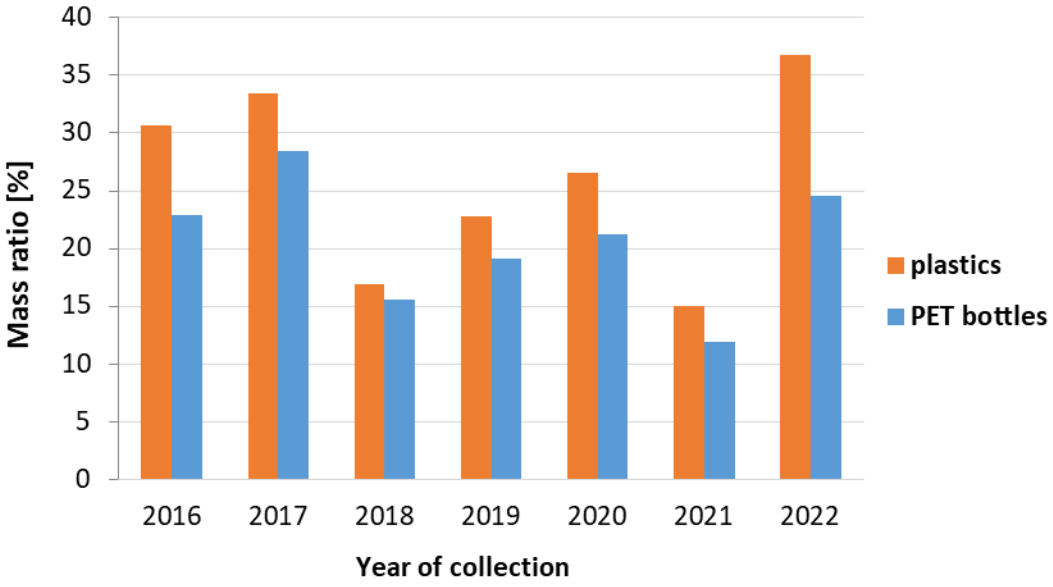

Figure S1: *The mass ratio of plastics and PET bottles in the riverine litter collected from the floodplains of Tisza River in Hungary in recent years*

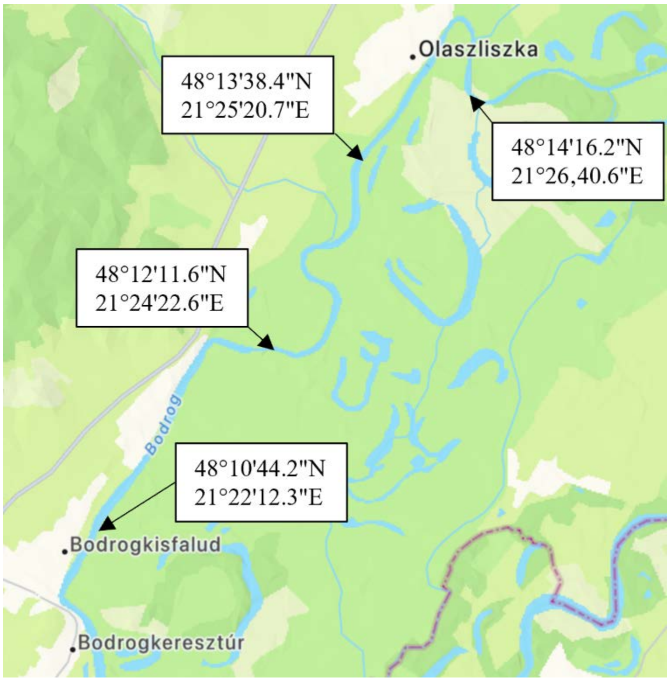

***Figure S2**: Coordinates of the locations where the analysed PET bottles were collected*

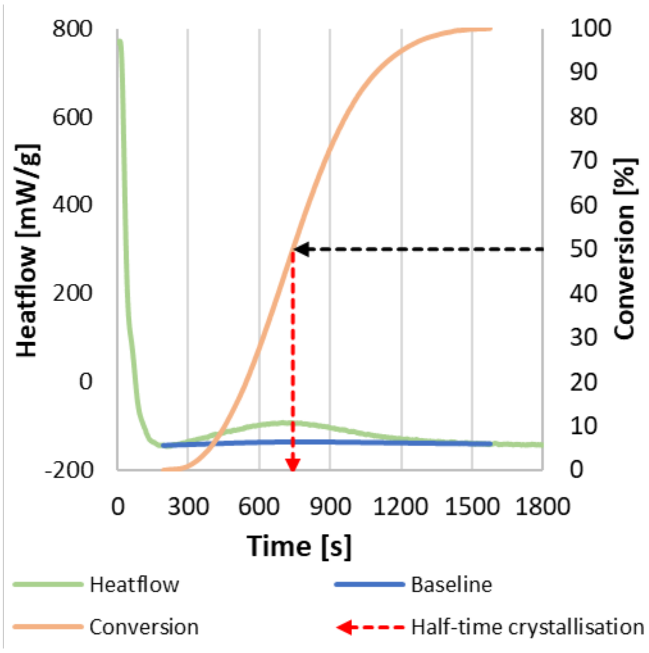

Figure S3: *Determination of the half-time crystallisation presented on the isothermal DSC curve of a sample collected from Bodrog River*

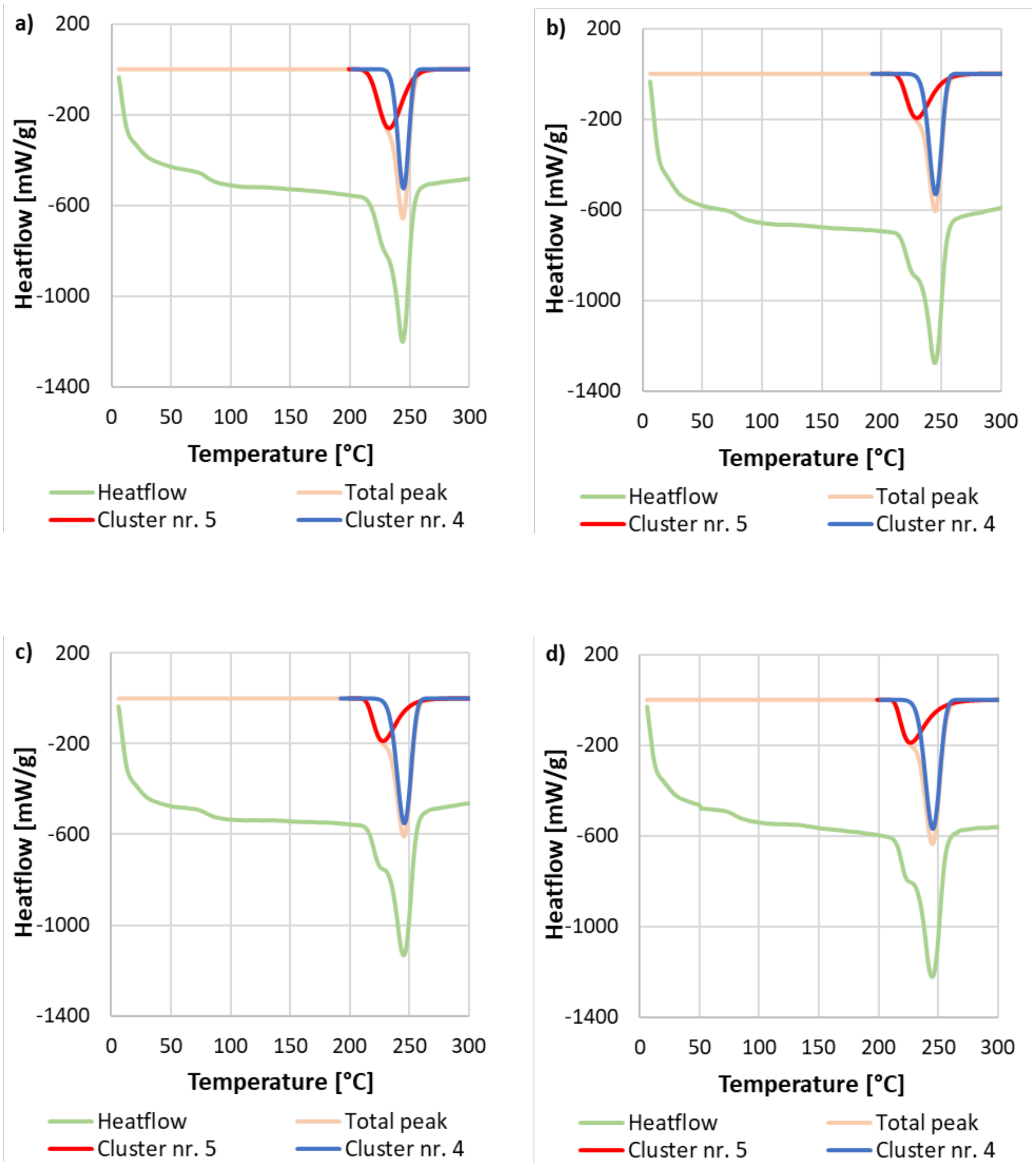

a)
b)
c)
d)
Heatflow [mW/g]
200
-200
-600
-1000
-1400
0
50
100
150
200
250
300
Temperature [°C]
Heatflow
Total peak
Cluster nr. 5
Cluster nr. 4

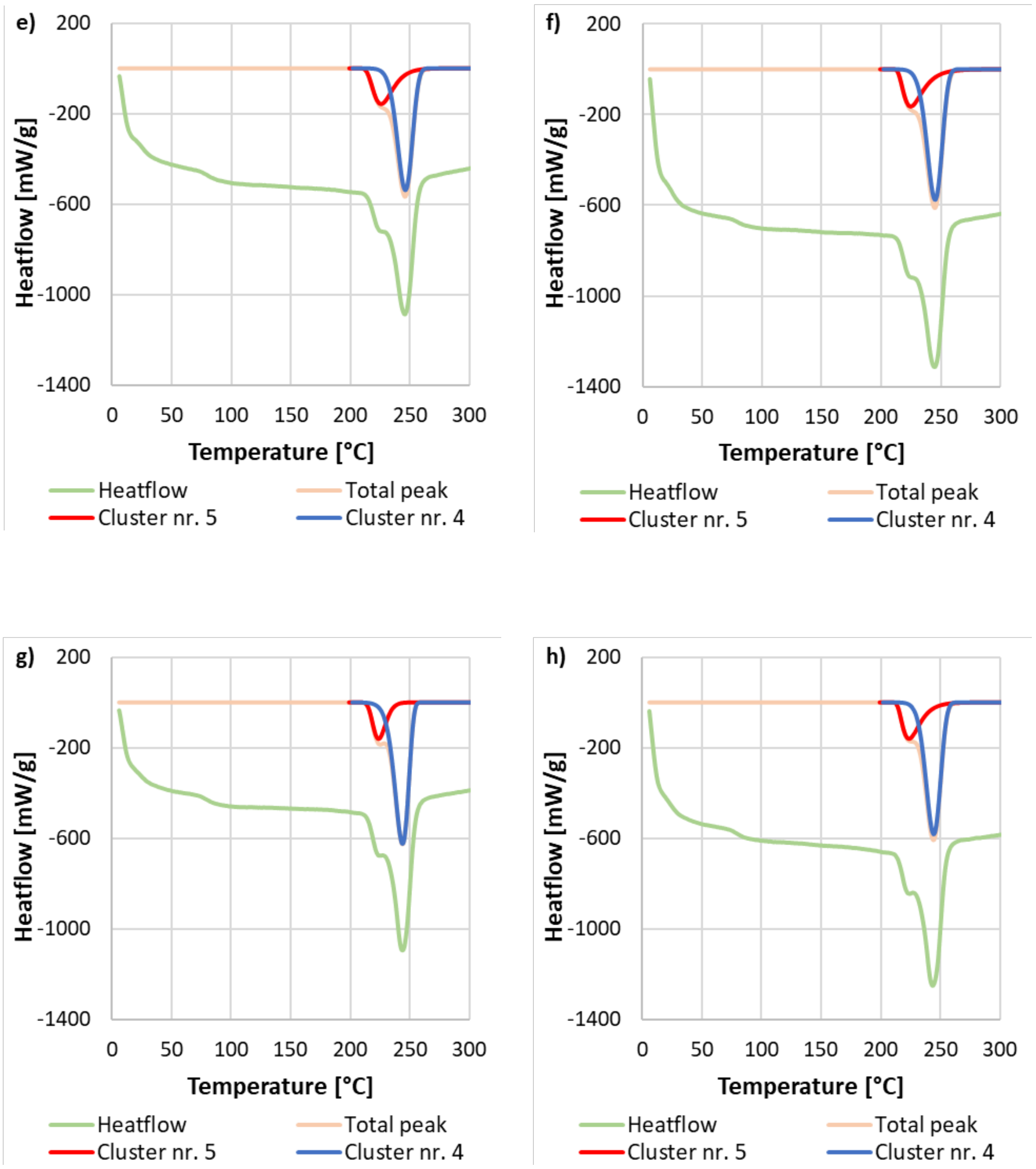

Figure S4: *DSC heating curves of PET bottles aged for (a) 0 h, (b) 168 h, (c) 336 h, (d) 504 h, (e) 672 h, (f) 840 h, (g) 1008 h and (h) 1176 h after the isothermal crystallization*

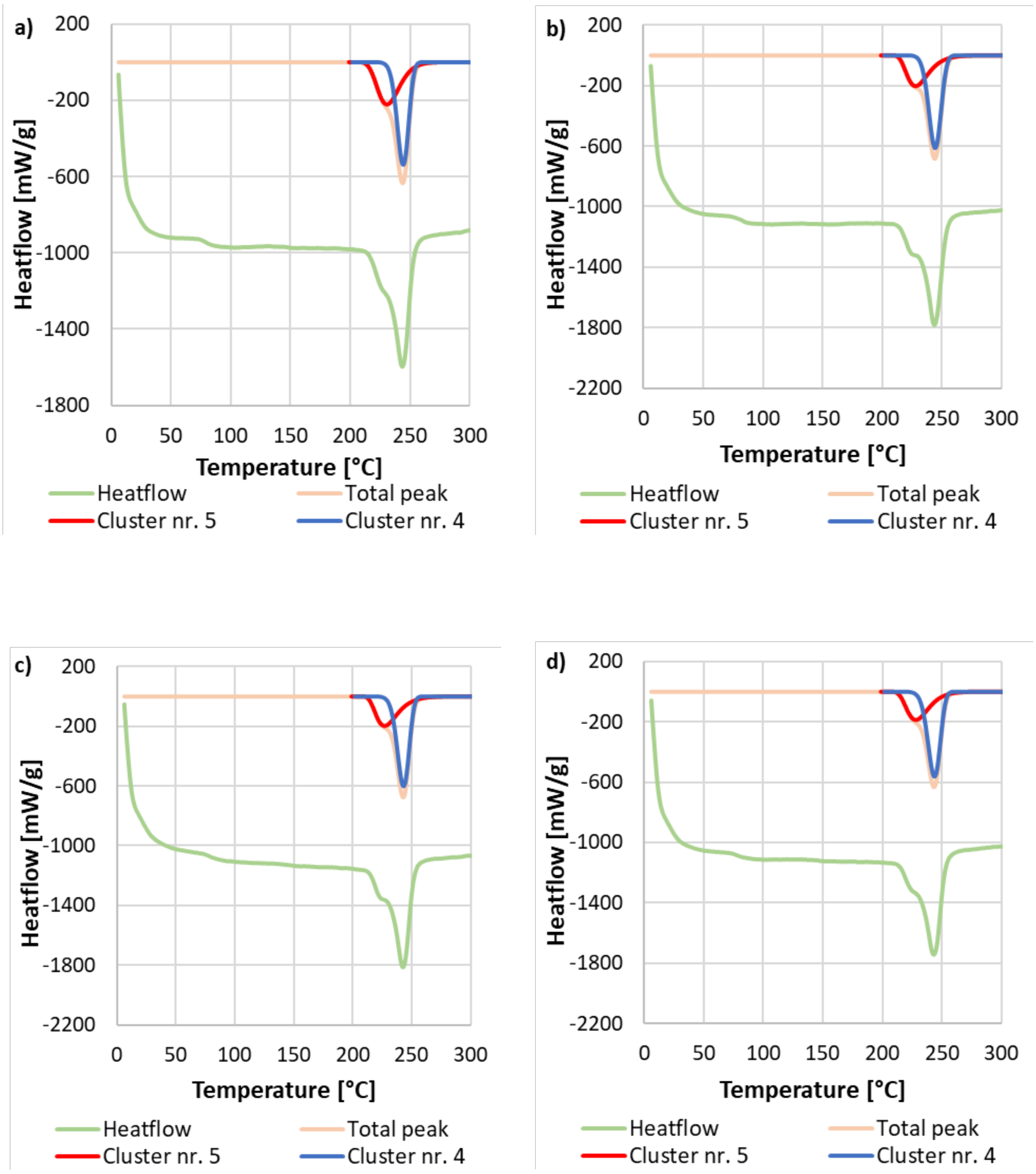

a)
b)
c)
d)
Heatflow [mW/g]
Temperature [°C]
200
-200
-600
-1000
-1400
-1800
-2200
0
50
100
150
200
250
300
Heatflow
Total peak
Cluster nr. 5
Cluster nr. 4

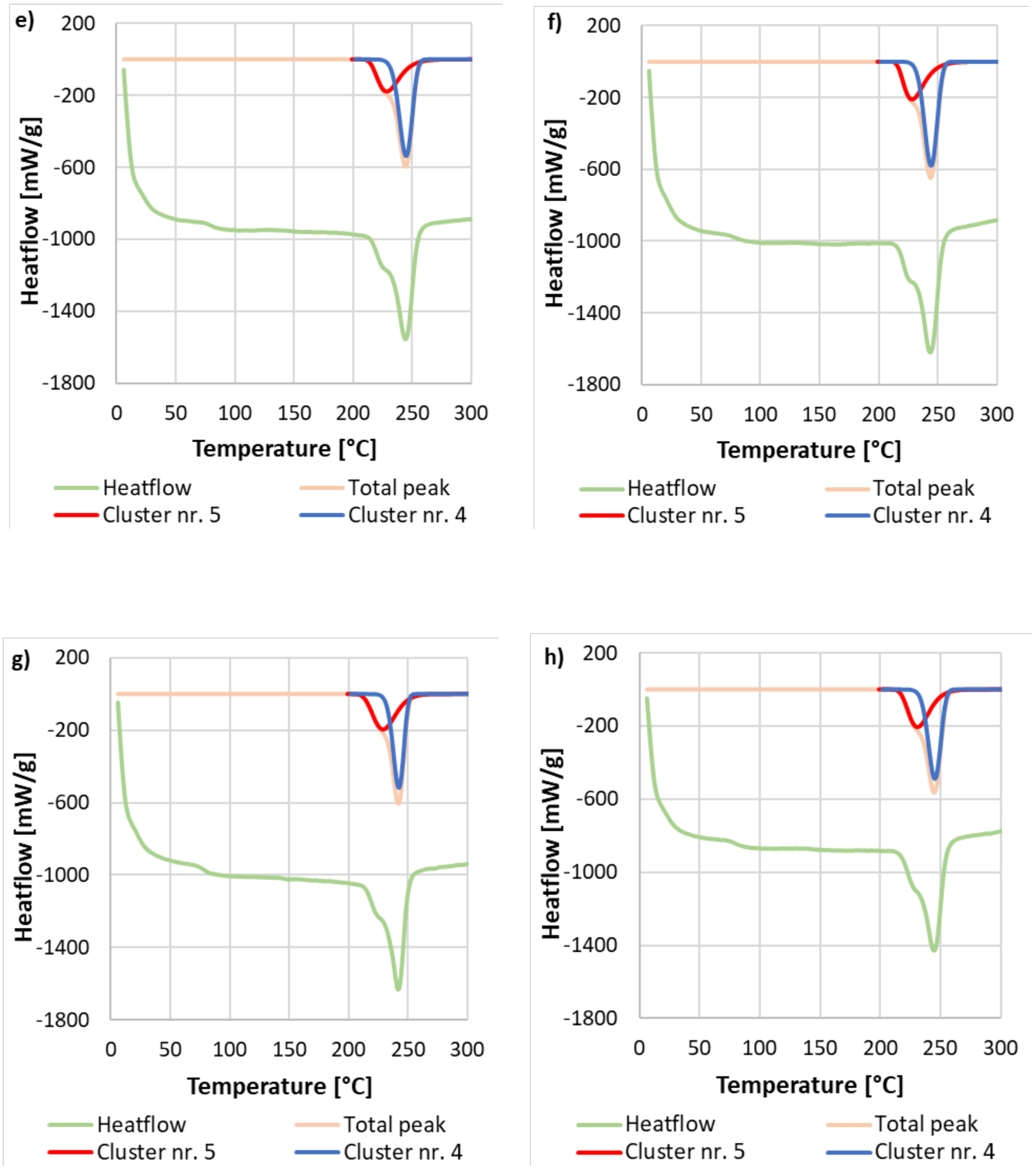
e)
f)
g)
h)
Heatflow [mW/g]
200
-200
-600
-1000
-1400
-1800
0
50
100
150
200
250
300
Temperature [°C]
Heatflow
Total peak
Cluster nr. 5
Cluster nr. 4

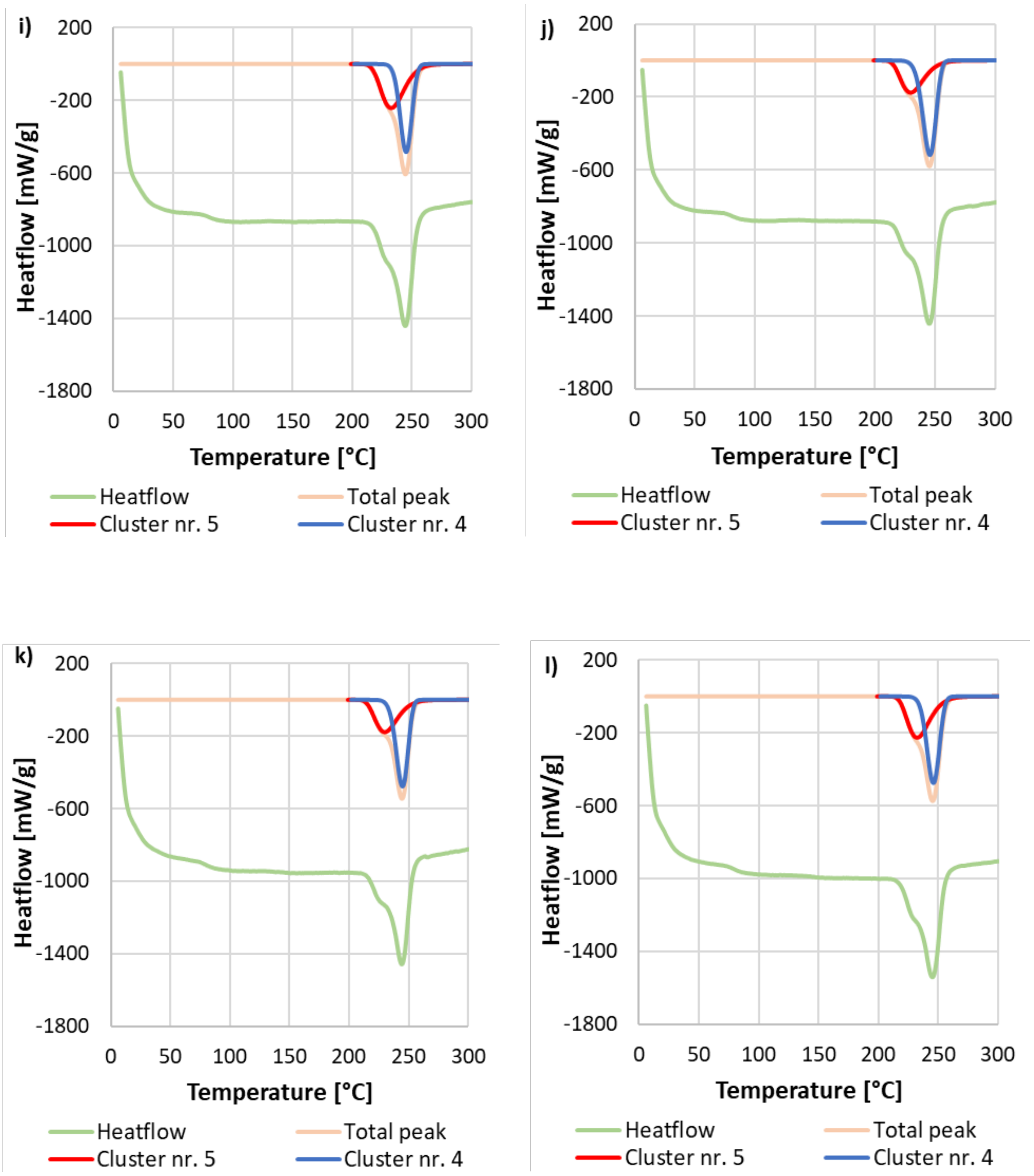
i)
Heatflow [mW/g]
Temperature [°C]
Heatflow
Total peak
Cluster nr. 5
Cluster nr. 4
j)
Heatflow [mW/g]
Temperature [°C]
Heatflow
Total peak
Cluster nr. 5
Cluster nr. 4
k)
Heatflow [mW/g]
Temperature [°C]
Heatflow
Total peak
Cluster nr. 5
Cluster nr. 4
l)
Heatflow [mW/g]
Temperature [°C]
Heatflow
Total peak
Cluster nr. 5
Cluster nr. 4

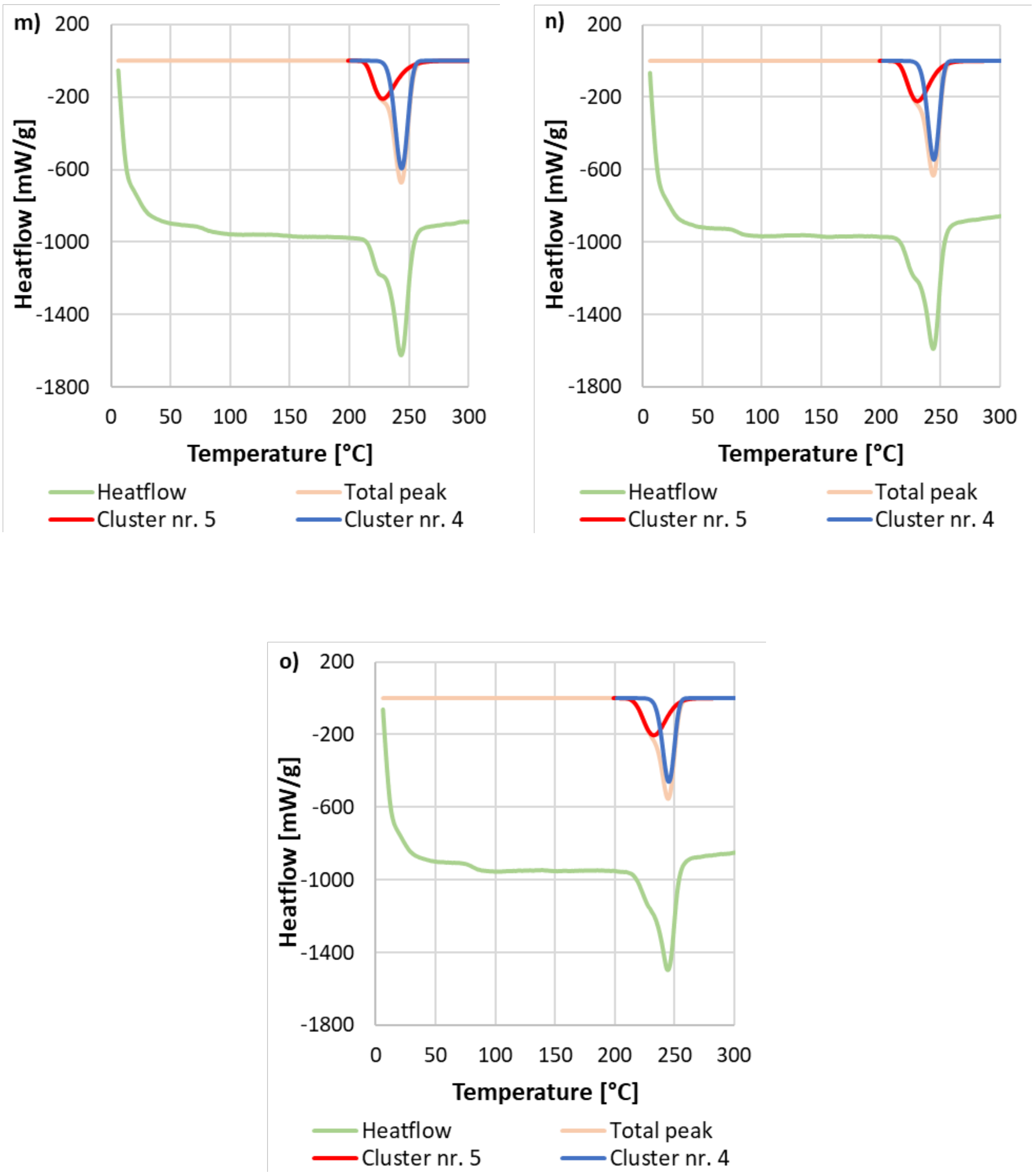

Figure S5: *(a-o) DSC heating curves of samples collected from Bodrog River after the isothermal crystallization*
